# New extended *(G’/G)*-expansion method to solve nonlinear evolution equation: the (3 + 1)-dimensional potential-YTSF equation

**DOI:** 10.1186/2193-1801-3-122

**Published:** 2014-03-05

**Authors:** Harun-Or- Roshid, M Ali Akbar, Md Nur Alam, Md Fazlul Hoque, Nizhum Rahman

**Affiliations:** Department of Mathematics, Pabna University of Science and Technology, Pabna, Bangladesh; Department of Applied Mathematics, University of Rajshahi, Rahjshahi, Bangladesh

**Keywords:** *(G’/G)*-expansion method, (3 + 1)-dimensional potential-YTSF equation, Traveling wave solutions, Homogeneous balance

## Abstract

In this article, a new extended (*G*′/*G*) -expansion method has been proposed for constructing more general exact traveling wave solutions of nonlinear evolution equations with the aid of symbolic computation. In order to illustrate the validity and effectiveness of the method, we pick the (3 + 1)-dimensional potential-YTSF equation. As a result, abundant new and more general exact solutions have been achieved of this equation. It has been shown that the proposed method provides a powerful mathematical tool for solving nonlinear wave equations in applied mathematics, engineering and mathematical physics.

## Introduction

The world around us is inherently nonlinear and nonlinear evolution equations (NLEEs) are extensively used to model the complex physical phenomena. The exact solutions of NLEEs play a crucial role in nonlinear science and engineering. Therefore, investigation of exact solutions of nonlinear partial differential equations has become a major concern for both mathematicians and physicists. One of the fundamental problems for these models is to obtain their traveling wave solutions. Therefore, the interest on finding traveling wave solution of NLEEs is increasing day by day and now it becomes a hot topic to the researchers. In recent years, many researchers who are interested in the nonlinear physical phenomena investigated exact solutions of NLEEs. They established many powerful and direct methods to comprehend the internal mechanisms of these physical phenomena. Some of the existing methods are, the Backlund transformation method (Miura, 1978; Wang and Wang, 2001), the Darboux transformation method (Matveev and Salle, 1991), the Riccati equation method (Cai et al., 2009), the tanh-function method (Fan, 2000; Wazwaz, 2004a), the Exp-function method (He and Wu, 2006), the sine-cosine method (Wazwaz, 2004b), the Frobenius integrable decomposition method (Ma et al., 2007), the rational function transformation method (Ma and Lee, 2009), the multiple Exp-function method (Ma and Zhu, 2012; Ma et al., 2010), the generalized bilinear transformation method (Ma, 2011), the Cole-Hopf transformation method (Ma and Fuchssteiner, 1996), the bilinear differential operator scheme (Ma, 2013), the homogeneous balance method (Fan and Zhang, 1998), the auxiliary equation method (Sirendaoerji, 2003), the Lie group transformation method (Olver, 1986) and so on.

Recently, Wang et al. (Wang et al., 2008) established a prolific method called the (*G*′/*G*)-expansion method. Applications of this method can be found in the works, Zayed and Gepreel (2008); Ozis and Aslan (2009); Kheiri and Jabbari (2010); Naher et al. (2011); Akbar et al. (2012a); Guo et al. (2010); Zayed and Al-Joudi (2010) and references therein for better comprehension. Then diverse group of researchers extended this method in different names like, extended (*G*′/*G*)-expansion method (Guo and Zhou, 2010), further extended (*G*′/*G*)-expansion method (Li et al., 2010), improved (*G*′/*G*)-expansion method (Zayed, 2011), generalized and improved (*G*′/*G*)-expansion method (Akbar et al., 2012b) etc.

In this article, following the above extensions of the (*G*′/*G*)-expansion method, we offer a scheme called the new extended (*G*′/*G*)-expansion method in which the solution is presented in the form  where *G* = *G*(*ξ*) satisfies the differential equation *G*″ + *μ G* = 0, *μ* ≠ 0. Using this method, we achieve several new traveling wave solutions of the (3 + 1)-dimensional potential-YTSF equation.

## The method

For the independent variables *x, y, z* and *t*, we consider the NLEEs in the following form
1

where *u* = *u*(*x*, *y*, *z*, *t*) is an unknown function and *F* is a polynomial in *u*(*x*, *y*, *z*, *t*) and its partial derivatives.

Consider the traveling wave transformation
2

where *V* is the speed of the traveling wave to be determined. The principal steps of the method are as follows:

**Step 1.** Using the traveling wave transformation (2), Eq. (1) can be converted into an ordinary differential equation (ODE):
3

where prime stands for ordinary derivative with respect to *ξ* and *Q* is a polynomial in *u* = *u*(*ξ*) and its derivatives.

**Step 2.** Assume that the solution of Eq. (3) can be expressed in the following form
4

where *G* = *G*(*ξ*) satisfies the differential equation
5

where *μ* ≠ 0, *σ* = ± 1 and *a*_*i*_, *b*_*i*_ (*i* = − *n*, ⋯, *n*), *λ* are constants to be determined.

**Step 3.** The value of *n* can be determined by balancing the highest-order derivative term with the nonlinear term of the highest order come out in the reduced equation (3).

**Step 4.** Inserting (4) into Eq. (3) and making use of Eq. (5) and then extracting all terms of powers of (*G*′/*G*)^*j*^ and  and setting each coefficient of them to zero yields an over-determined system of algebraic equations. Solving this system of algebraic equations for *a*_*i*_, *b*_*i*_ (*i* = − *n*, ⋯, *n*) and *λ*, *V*, we obtain the value of the unknown parameters.

**Step 5.** The substitution *y* = (*G*′/*G*) transforms the linear Eq. (5) into the following Riccati equation:
6

where *y* = (*G*′/*G*). The general solutions of the Riccati Eq. (6) are well-known (see Ma and Fuchssteiner, 1996) and are given below:
78

Inserting the values of *a*_*i*_, *b*_*i*_ (*i* = − *n*, ⋯, *n*),  *λ*, *V* and (7) and (8) into Eq. (4), we obtain abundant traveling wave solutions of Eq. (1). In Ref. Ma and Fuchssteiner (1996), a solution for the condition *μ* = 0 also presented. But, since in step 5, we assumed *μ* ≠ 0, this solution is not presented here. If *μ* = 0, the assumed solution (4) will collapse.

## Application of the method

To show the efficiency of the proposed method, we consider the (3 + 1)-dimensional potential-YTSF equation
9

Under the traveling wave transformation provided in Eq. (2), Eq. (9) will be transformed to an ODE and integrating once, we obtain
10

where *K* is an integration constant. The balance of the highest-order derivative term *u*‴ and nonlinear term of the highest order *u*′^2^ in Eq. (10) yields *n* = 1. Therefore, according to our preamble, the solution of Eq. (11) is
11

where *G* = *G*(*ξ*) satisfies Eq. (5). Substituting Eq. (11) and Eq. (5) into Eq. (10) and collecting the terms of like powers of (*G*′/*G*)^*j*^ and , and setting them to zero, we obtain an over-determined system of algebraic equation that consists of twenty-five equations (the equations are omitted here for minimalism). Solving this over-determined system of algebraic equations with the assist of Maple, we obtain the following solutions:

Case-1:


When *μ* > 0, using (7) into solution Eq. (11), we obtain
12

and
13

where *ξ* = *x* + *y* + *z* − *V t* and *V* = *μ* − 3/4.

Again, when *μ* < 0, using (8) into solution Eq. (11), we obtain
14

and
15

Case-2:


When *μ* > 0, using (7) into solution Eq. (11), we obtain
1617

where *ξ* = *x* + *y* + *z* − *V t*,  *V* = *μ* − 3/4.

And when *μ* < 0, using (8) into solution Eq. (11), we obtain
1819

where *ξ* = *x* + *y* + *z* − *V t*,  *V* = *μ* − 3/4.

Case-3:


When *μ* > 0, using (7) into solution Eq. (11), we obtain
20

where *ξ* = *x* + *y* + *z* − *Vt*, *V* = *μ* − 3/4 and *i* = 1 2.

And when *μ* < 0, using (8) into solution Eq. (11), we obtain
21

where *ξ* = *x* + *y* + *z* − *Vt*, *V* = *μ* − 3/4 and *i* = 1 2.

Case-4:


When *μ* > 0, using (7) into solution Eq. (11), we obtain
22

where *ξ* = *x* + *y* + *z* − *Vt*, *V* = 4*μ* − 3/4 and *i* = 1 2.

And when *μ* < 0, we obtain
23

where *ξ* = *x* + *y* + *z* − *Vt*, *V* = 4*μ* − 3/4 and *i* = 1 2.

Case-5:


When *μ* > 0, we obtain
24

where *ξ* = *x* + *y* + *z* − *Vt*, *V* = 4*μ* − 3/4 and *i* = 1 2.

And when *μ* < 0, we obtain
25

where *ξ* = *x* + *y* + *z* − *Vt*, *V* = 4*μ* − 3/4 and *i* = 1 2.

## Comparison

It is worth declaring that one of our obtained solutions is in good agreement with already published results which is presented in the following Table 1.

**Table 1:** **Comparison between Ma et al. (** 2010 **) solutions and our solutions**

Ma et al. ( 2010 ) solutions	Obtained solutions
i. If *b*_0_ = 0 and *b*_1_ = 1, solutions Eq. (3.5) becomes:	i. If *a*_0_ = 0, *k*_0_ = − 2*μ*, *a*_1_ = − 2*μλ*, *f*_1 *i*_(*ξ*) = *e* ^*ξ*^ then the solution of equation (20) is

Moreover, in this article, abundant traveling wave solutions of the (3 + 1)-dimensional potential YTSF equation is constructed by applying the proposed method. Solutions obtained by means of the new extended (*G*′/*G*)-expansion method are distinct from the solutions obtained by Ma et al. (2010). The solutions (12)-(19) and (21)-(25) were not obtained by Ma et al. (2010). On the other hand, the auxiliary equation used in this article is different, so obtained solutions is also different.

### Remark

The solutions obtained in this article have been checked by putting them back into the original equation and found correct.

## Discussions

The advantages and validity of the method over extended (*G*′/*G*)-expansion method have been discussed in the following.

### Advantages

The vital advantage of the new extended (*G*′/*G*)-expansion method over the basic (*G*′/*G*)-expansion method is that the method provides more general and large amount of new exact traveling wave solutions with several free parameters. The exact solutions have its great importance to expose the inner mechanism of the complex physical phenomena. Apart from the physical application, the close-form solutions of nonlinear evolution equations assist the numerical solvers to compare the accuracy of their results and help them in the stability analysis.

### Validity

Zayed and El-Malky (2010) investigated solutions of the (3 + 1)-dimensional potential-YTSF equation by using extended (*G*′/*G*)-expansion method. They got four set of solutions of the algebraic equations and the solutions of the potential-YTSF equation are given in Eqs. (A.1)-(A.8) (see Appendix). On the other hand in this article we obtained five set of solutions of the algebraic equations. It is observed that using a simple transformation then Zayed and El-Malky’s (2010) solutions (A.1) and (A.2) are identical to our solutions (12)-(15) and solutions (22) and (23) are similar to (A.7) and (A.8). Now if we set *λ* = 0, then solutions (16) and (17) are identical to (A.4), solutions (18) and (19) are identical to (A.3), solution (20) is identical to (A.6) and solution (21) is identical with (A.5). If *λ* ≠ 0, solutions (16)-(21) are different from the solutions (A.3)-(A.6). Therefore, we can make a decision that solutions (A.3)-(A.6) are particular cases of solutions (16)-(21). Further we obtain solutions (24) and (25) which are not obtained by Zayed and El-Malky (2010).

## Conclusion

A new extended (*G*′/*G*)-expansion method has been established in this article to search for exact traveling wave solutions for nonlinear evolution equations. The method is direct, straightforward and easy to implement. In order to illustrate the validity and advantages of the algorithm, we apply the method to the (3 + 1)-dimensional potential-YTSF equation and abundant traveling wave solutions are achieved. The solutions are obtained in the form of trigonometric and hyperbolic functions. The performance this method is effective and productive. The method might be applied to solve different nonlinear PDEs which frequently arise in mathematical physics, engineering sciences and many scientific real time application fields.

## Appendix

### Appendix: Zayed and El-Malky’s solutions (2010)

Zayed and El-Malky (2010) established exact solutions of the well-known (3 + 1)-dimensional potential-YTSF equation by using the extended (*G*′/*G*)-expansion method. They found the following solutions
A.1

where *ξ* = *x* + *y* + *z* + (3/4 − *μ*/4)*t*A.2

where *ξ* = *x* + *y* + *z* + (3/4 − *μ*/4)*t*A.3

where *ξ* = *x* + *y* + *z* + (3/4 − *μ*)*t*A.4

where *ξ* = *x* + *y* + *z* + (3/4 − *μ*)*t*A.5

where *ξ* = *x* + *y* + *z* + (3/4 − *μ*)*t*A.6

where *ξ* = *x* + *y* + *z* + (3/4 − *μ*)*t*A.7

where *ξ* = *x* + *y* + *z* + (3/4 − 4*μ*)*t*A.8

where *ξ* = *x* + *y* + *z* + (3/4 − 4*μ*)*t*.
